# Antibodies against interleukin-10 receptor reduce IL-6 and TNF-α levels and increase TGF‐β levels in patients with severe fever with thrombocytopenia syndrome virus and SARS-CoV-2 infection

**DOI:** 10.3389/fimmu.2026.1828107

**Published:** 2026-06-25

**Authors:** Su Yeon Kang, Jeong Rae Yoo, EunJin Bae, Joowan Kim, Yejin Park, Misun Kim, Miyeon Kim, Hyo-Jin Ro, Daehee Hwang, Jeong-Yeon Lee, Dongcharn Cho, Huy Chau Nguyen, Hoai Jaclyn Hallam, Sang Taek Heo, Nam-Hyuk Cho, Kyung-Mi Lee, Andrew G. Letizia, Keun Hwa Lee

**Affiliations:** 1 Hanyang University College of Medicine and Intercollege, Seoul, Republic of Korea; 2 Department of Internal Medicine, Jeju National University College of Medicine, Jeju, Republic of Korea; 3 Department of Microbiology and Immunology, Seoul National University College of Medicine, Seoul, Republic of Korea; 4 Department of Biochemistry, Korea University College of Medicine, Seoul, Republic of Korea; 5 School of Biological Science, Seoul National University, Seoul, Republic of Korea; 6 US Naval Medical Research Unit INDO PACIFIC, Singapore, Singapore

**Keywords:** antibodies against interleukin-10 receptor, IL-6, SARS-CoV-2, severe fever with thrombocytopenia syndrome, TGF‐β, TNF-α

## Abstract

Severe fever with thrombocytopenia syndrome virus (SFTSV) and severe acute respiratory syndrome coronavirus 2 (SARS-CoV-2) can cause severe, often fatal, disease characterized by hyperinflammation and features of a cytokine storm. Hyperproduction of both IL-10 and IL-6 and low TGF-β production can generate a cytokine storm, with IL-10 playing a particularly important role. To investigate the role of IL‐10 in patients with SFTS, we analyzed the phenotypes of macrophages, cytokines, and signaling pathways in patients with mild to fatal SFTS and found that the population of HLA-DR^+^CD86^+^ macrophages was increased, the population of CD163^+^CD206^+^ macrophages was decreased, the levels of IL-10 (*p* < 0.0001), IL-6 (*p* < 0.0001), TNF-α (*p=*0.1056), and CCL1 (*p=*0.1533) were increased, TGF-β (*p=*0.0104) was increased, and Smad3 and P-Smad3 were highly expressed in patients with fatal SFTS. We also investigated the role of IL‐10 in THP‐1-derived macrophages infected with SFTSV or SARS‐CoV‐2, treated with lipopolysaccharide (LPS), or treated with serum from patients with fatal SFTS. We found that blocking IL‐10 signaling can decrease the population of HLA-DR^+^CD86^+^ cells, increase the population of CD163^+^CD206^+^ cells, reduce IL‐6 and TNF-α production, increase TGF‐β production and induce the expression of Smad3 and P-Smad3 in SFTSV- and SARS‐CoV‐2‐infected and LPS‐induced THP-1 cells. Additionally, IL-10 receptor blockade can reduce IL-10 and IL‐6 production in THP-1 cells treated with serum from patients with fatal SFTS. Therefore, we suggest that HLA-DR^hi^CD86^hi^ macrophages may contribute to pathological activity and that CD163^hi^CD206^hi^ macrophages may play a critical role in the protection of effector functions against SFTSV and SARS-CoV-2 infection. IL-10 could serve as a prognostic target, and antibodies against the IL-10 receptor could represent a potential immune-based intervention against a cytokine storm in patients with fatal SFTS and severe/critical COVID-19.

## Introduction

Severe fever with thrombocytopenia syndrome (SFTS), a new tick-borne viral disease with a high mortality rate, was first reported in China in 2009, South Korea in 2010, Japan in 2013, Vietnam in 2017, and Pakistan and Thailand in 2020 (1–6). Severe fever with thrombocytopenia syndrome virus (SFTSV, officially named *Dabie bandavirus*) and severe acute respiratory syndrome coronavirus 2 (SARS-CoV-2) can cause severe, often fatal, disease characterized by hyperinflammation and a cytokine storm with rapid and prolonged systemic elevation of interleukin 10 (IL-10) and IL-6 (1, 2, 7–13).

SFTSV and SARS-CoV-2 are not only a risk to the local populations, but also to transient populations including travelers and military personnel who may be visiting, working, conduct training exercises or deployments in the affected regions. By understanding the cellular immune response, we can better inform risk assessments and mitigations to protect local and military populations.

We reported that systemic hyperproduction of both IL-10 and IL-6 and low systemic production of transforming growth factor‐β (TGF-β) can generate a cytokine storm in patients with fatal severe fever with thrombocytopenia syndrome (SFTS) and severe and critical coronavirus disease 2019 (COVID-19) and suggested that IL-10 may play a pathological role in the severity of SFTS and COVID-19 (7, 8, 10–13).

Macrophages are the major innate immune cells involved in infection and inflammation, and they are the first line of defense against SFTSV and SARS-CoV-2. Macrophage activation syndrome (MAS), a form of cytokine release syndrome (CRS), also known as a cytokine storm, is observed in patients with fatal SFTS and severe and critical COVID-19 (12, 14).

The Janus kinase 1/signal transducer and activator of transcription 3 (JAK1/STAT3) pathway is a rapid membrane-to-nucleus signaling system that regulates gene expression in response to cytokines, leading to the production of IL-10 and IL-6 in macrophages, and the connections among IL-6, JAK, and STAT3 are closely related to the severity of COVID-19 (12).

Suppressor of Mothers Against Decapentaplegic (Smad) 2 and 3 are key components of the TGF-β signaling pathway, with a critical influence on macrophage polarization, and the link between high levels of Smad3 activity and increased IL-10 levels has been documented (15).

Therefore, we studied the phenotypes of macrophages and the JAK1/STAT3 and Smad2/3 signaling pathways in patients with SFTS ranging in severity from mild to fatal and revealed that the population of HLA-DR^hi^ (MHC class II) CD86^hi^ macrophages, which produce IL-10, IL-6, and chemokine (C-C motif) ligand 1 (CCL1), and the population of CD163^hi^ (a scavenger receptor for hemoglobin) CD206^hi^ (the mannose receptor) macrophages were decreased in patients with fatal SFTS. In addition, Smad3 and its phosphorylated and activated form (P-Smad3) were highly expressed in patients with fatal SFTS.

When we blocked IL‐10 signaling using antibodies against the IL‐10 receptor *in vitro* in human macrophages under different conditions-SFTSV infection, SARS-CoV-2 infection, and LPS induction-the population of HLA-DR^+^CD86^+^ macrophages decreased, the population of CD163^+^CD206^+^ macrophages increased, and Smad3 and P-Smad3 were highly expressed.

We also found that the levels of IL-10, IL-6, tumor necrosis factor (TNF-α), and CCL1 were elevated in patients with fatal SFTS. Blocking IL‐10 signaling with antibodies against the IL‐10 receptor can reduce IL‐6 and TNF-α production and increase TGF‐β production in SFTSV- and SARS‐CoV‐2‐infected and LPS‐induced human macrophages, and blocking IL‐10 signaling with antibodies against the IL‐10 receptor can reduce IL-10 and IL‐6 production in human macrophages treated with serum from patients with fatal SFTS (8, 13).

Therefore, we suggest that Smad3^+^HLA-DR^hi^CD86^hi^ macrophages, which produce IL-10, IL-6, and TNF-α, as well as CCL1, may contribute to pathological activity and that Smad3^+^CD163^hi^CD206^hi^ macrophages, which secrete TGF-β, may play critical roles in the protective effector functions during SFTSV and SARS-CoV-2 infection; moreover, IL-10 could serve as a prognostic target, and antibodies against the IL-10 receptor are potential immune-based interventions against fatal SFTS and critical COVID-19 (8, 11, 13, 16–18).

## Materials and methods

### SFTS patients

SFTS patients were recruited from May 2013 to July 2024. During the study period, 86 patients were confirmed to be positive for partial small (S) and large (L) SFTSV RNA segments using real-time RT–PCR and were analyzed in the present study (**Supplementary Table 1**) (19).

### Analyses of IL-10, IL-6, TNF-α, CCL1, and TGF-β in SFTS patients

IL-10, IL-6, and TNF-α levels in SFTS patients were measured using human Th1/Th2/Th17 CBA kits (BD Bioscience, San Diego, CA, USA) according to the manufacturer’s instructions, with minor modifications. Sample acquisition was performed with a FACSCanto II flow cytometer, and the data were analyzed using FCAP Array software version 3.0 (BD Bioscience). All the statistical analyses were performed using SPSS 22.0 (SPSS, an IBM Company). TGF-β levels in serum samples were measured using a TGF-β-1 Human ELISA Kit (Thermo Fisher Scientific), and CCL1 levels in serum samples were measured using a CCL1 Human ELISA Kit (Thermo Fisher Scientific) according to the manufacturer’s protocols. To compare the mean differences between patients with fatal and nonfatal SFTS disease, we typically used a two-sample t test. When using this method, we checked assumptions such as normality, equal variance, and independence. When these assumptions were not met, we used a nonparametric two-sample t test called the Wilcoxon–Mann–Whitney test (7, 8). *P* values<0.05 indicated statistical significance.

### Imaging flow cytometry

PE-conjugated anti-human HLA-DR (cat. 307606), FITC-conjugated anti-human CD86 (cat. 374204), APC-conjugated anti-human CD163 (cat. 333610), and Alexa Fluor 700-conjugated anti-human CD206 (cat. 321132) antibodies from BioLegend were used according to the manufacturer’s instructions, with minor modifications. Sample acquisition was performed using a FACSCanto II flow cytometer, and the data were analyzed using FlowJo software version 10.9 (BD Bioscience).

### Western blotting

Total protein was extracted from THP-1 cells with RIPA lysis buffer (IBS-BR002; iNtRON Biotechnology). The proteins were electrophoresed on an 8% SDS–PAGE gel and transferred to mixed cellulose ester transfer membranes (HATF00010; Merck). After blocking, the membranes were incubated with primary antibodies at 4 °C overnight. The antibodies used in this study included rabbit polyclonal antibodies against Jack1 (3332; Cell Signaling Technology), p-Jack1 (3331; Cell Signaling Technology), and Smad2/3 (3102; Cell Signaling Technology); rabbit monoclonal antibodies against p-Smad2/3 (8828; Cell Signaling Technology); and mouse monoclonal antibodies against Stat3 (SC-8019; Santa Cruz Biotechnology), p-Stat3 (SC-8059; Santa Cruz Biotechnology), and β-actin (3700; Cell Signaling Technology). Following incubation with primary antibodies, the membranes were incubated with HRP-conjugated goat anti-mouse IgG (H+L) (31430; Invitrogen) or goat anti-rabbit IgG (H+L) (31460; Invitrogen) secondary antibodies. The protein expression levels were visualized using a ChemiDoc XRS+ imaging system (Bio-Rad).

### THP-1 cells, SFTSV, SARS-CoV-2, and IL‐10RA poly- and monoclonal antibodies

The human monocytic cell line THP-1 (ATCC TIB-202) was used to model macrophages. THP-1 cells were cultured in RPMI-1640 medium (Gibco) supplemented with 10% fetal bovine serum (FBS) (Gibco), 1% penicillin–streptomycin (Gibco), 200 mM L-glutamine (Gibco) and 55 mM 2-mercaptoethanol (Gibco) and kept in a humidified 5% CO_2_ incubator at 37 °C. THP-1 cells were differentiated into a macrophage phenotype at a density of 2–4 × 10^5^ cells/mL in 100 mm cell culture dishes (Corning Ins.), treated with 100 ng/mL phorbol myristate acetate (PMA) (Sigma-Aldrich) for 24 hours (h), and then washed and suspended in culture medium without PMA.

SFTSV (GenBank accession no. MN329148-MN329150) was isolated from a Korean SFTS patient. The virus was propagated and titrated in Vero E6 cells (ATCC CRL-1586), which were subsequently cultured in Dulbecco’s modified Eagle’s medium (DMEM) (Gibco) supplemented with 10% FBS (8).

SARS-CoV-2/BA.1.1 was isolated from a nasopharyngeal swab taken from a patient with COVID-19. The virus was propagated and titrated in Vero E6 cells (ATCC CRL-1586), which were subsequently cultured in Dulbecco’s modified Eagle’s medium (DMEM) (Gibco) supplemented with 2% FBS and 1% penicillin-streptomycin (Gibco) (8).

The polyclonal and monoclonal antibodies against human IL-10RA used in the blocking experiments were purchased from St John’s Laboratory (cat. STJ24158) and developed in this study, respectively (**Table 1**).

**Table 1:** Comparison of IL-10, IL-6, TNF-α, CCL1, and TGF-β concentrations between media only, LPS only, and LPS with IL-10RA monoclonal antibodies.

Sample	IL-10	IL-6	TNF-α	CCL1	TGF-β
Media only	0.92	0.00	0.00	0.00	56.88
LPS only	256.47	3992.41	8710.25	1186.12	129.75
LPS with IL-10RA monoclonal antibodies	133.24	1777.82	3759.85	581.26	139.88

Unit: pg/mL.

### SFTSV-infected human macrophages were used to investigate the role of IL-10 receptor blockade

To investigate the role of IL-10 receptor blockade in SFTSV-infected THP-1 cells, THP-1 cells were infected with SFTSV at an MOI of 1, and SFTSV-infected THP-1 cells were divided into different treatment groups: untreated THP‐1 cells as the negative control group; SFTSV-infected THP-1 cells as the positive control group; and SFTSV-infected THP-1 cells treated with IL-10RA polyclonal antibodies (10 μg/mL) (Invitrogen). The three treatment groups were incubated for 12, 24 and 48 h, and the levels of IL-10, IL-6, and TNF-α were measured using human Th1/Th2/Th17 CBA kits (BD Bioscience) according to the manufacturer’s instructions, with minor modifications (7, 8). Sample acquisitions were performed with a FACSCanto II flow cytometer and analyzed with FCAP Array software version 3.0 (BD Bioscience). TGF-β levels in the collected supernatants were measured using a TGF-β-1 Human ELISA Kit (Thermo Fisher Scientific) according to the manufacturer’s protocols (8). CCL1 levels in the collected supernatants were measured using a CCL1 Human ELISA Kit (Thermo Fisher Scientific) according to the manufacturer’s protocols.

### Assessment of the role of IL-10 receptor blockade in SARS-CoV-2-infected human macrophages

To investigate the role of IL-10 receptor blockade in SARS-CoV-2-infected THP-1 cells, THP-1 cells were infected with SARS-CoV-2 at an MOI of 1, and SARS-CoV-2-infected THP-1 cells were divided into three treatment groups: untreated THP‐1 cells as the negative control group, THP-1 cells infected with SARS-CoV-2, and SARS-CoV-2-infected THP-1 cells treated with IL‐10RA polyclonal antibodies (10 μg/mL) (St John’s Laboratory cat. STJ24158). The three treatment groups were stimulated for 12, 24, and 48 h, and the levels of IL-10, IL-6, and TNF-α in the collected supernatants were measured using human Th1/Th2/Th17 CBA kits (BD Bioscience) according to the manufacturer’s instructions, with minor modifications (7, 8). Sample acquisitions were performed with a FACSCanto II flow cytometer and analyzed with FCAP Array software version 3.0 (BD Bioscience). TGF‐β levels in the collected supernatants were measured using a TGF‐β‐1 Human ELISA Kit (Thermo Fisher Scientific) according to the manufacturer’s protocols (3). CCL1 levels in the collected supernatants were measured using a CCL1 Human ELISA Kit (Thermo Fisher Scientific) according to the manufacturer’s protocols.

### Investigation of the role of IL-10 receptor blockade in LPS-induced human macrophages

THP-1 cells were divided into two treatment groups: THP-1 cells treated with LPS (2 μg/mL) (Sigma-Aldrich) as the control group and cells treated with LPS (2 μg/mL; Sigma-Aldrich, St Louis, USA) and IL-10RA polyclonal antibodies (10 μg/mL) (Invitrogen).

The two treatment groups were stimulated for 12, 24 and 48 h, and the levels of IL-10, IL-6, and TNF-α in the collected supernatants were measured using human Th1/Th2/Th17 CBA kits (BD Bioscience) according to the manufacturer’s instructions, with minor modifications (7, 8). Sample acquisitions were performed with a FACSCanto II flow cytometer and analyzed with FCAP Array software version 3.0 (BD Bioscience). TGF-β levels in the collected supernatants were measured using a TGF-β-1 Human ELISA Kit (Thermo Fisher Scientific) according to the manufacturer’s protocols^3^. CCL1 levels in the collected supernatants were measured using a CCL1 Human ELISA Kit (Thermo Fisher Scientific) according to the manufacturer’s protocols.

### Serum from patients with fatal SFTS-induced human macrophages were used to investigate the role of IL-10 receptor blockade

To investigate the role of IL-10 receptor blockade in the serum of patients with fatal SFTS, THP-1 cells were divided into two treatment groups: THP-1 cells treated with serum from patients with fatal SFTS and cells treated with serum and IL-10RA monoclonal antibodies (10 μg/mL) (**Tables 1**, **2**).

**Table 2:** Comparison of IL-10, IL-6, TNF-α, CCL1, and TGF-β concentrations between serum from patients with fatal SFTS and serum from patients with fatal SFTS with IL-10RA monoclonal antibodies.

SFTS patients	Condition	Time (Hours)	IL-10	IL-6	TNF-α	CCL1	TGF-β
JP-18-1011	Serum only	24	9.54	526.37	11.60	390.63	129.35
JP-17-0608			7.55	10544.19	0.00	257.27	112.18
JP-19-0713			7.90	130.50	0.00	214.31	197.17
JP-18-1011	Serum with IL-10RA monoclonal antibodies	24	8.47	181.35	10.76	184.87	53.17
JP-17-0608			5.09	8772.32	0.18	252.75	43.66
JP-19-0713			1.50	82.37	0.00	295.69	59.41

Unit: pg/mL.

The two treatment groups were stimulated for 24 h, and the levels of IL-10, IL-6, and TNF-α in the collected supernatants were measured using human Th1/Th2/Th17 CBA kits (BD Bioscience) according to the manufacturer’s instructions, with minor modifications. Sample acquisitions were performed with a FACSCanto II flow cytometer and analyzed with FCAP Array software version 3.0 (BD Bioscience). TGF-β levels in the collected supernatants were measured using a TGF-β-1 Human ELISA Kit (Thermo Fisher Scientific) according to the manufacturer’s protocols. CCL1 levels in the collected supernatants were measured using a CCL1 Human ELISA Kit (Thermo Fisher Scientific) according to the manufacturer’s protocols.

## Results

### Macrophages were affected in a patient with fatal SFTS: The population of Smad3^+^HLA-DR^hi^CD86^hi^ macrophages and serum levels of IL‐10, IL‐6, and CCL1 were high in a patient with fatal SFTS

**Figure 1:**
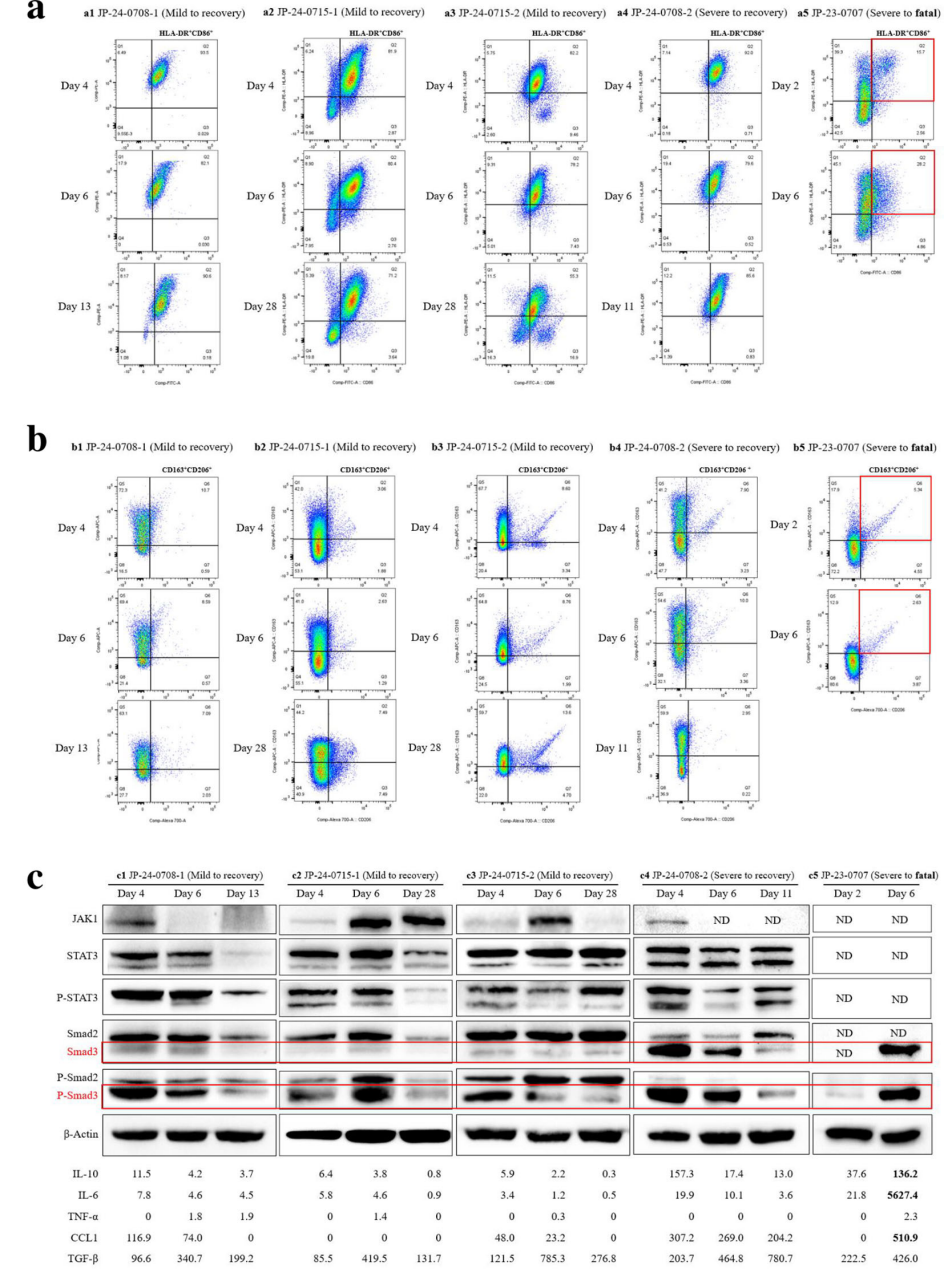
Multicolor flow cytometry analysis of HLA-DR and CD86 **(a1** to **a5)** and CD163 and CD206 **(b1** to **b5)** and western blotting **(c1** to **c5)** in SFTS patients (mild to fatal). The clinical characteristics of the SFTS patients are shown in **Supplementary Table 4**. SFTS, severe fever with thrombocytopenia syndrome.

We examined the phenotype of macrophages; the levels of IL‐10, IL‐6, CCL1, and TGF-β1; and the expression of JAK1/STAT3/Smad2/3 in SFTS patients with mild, moderate, severe, and fatal disease (**Supplementary Table 2**). The number of HLA-DR^+^CD86^+^ macrophages was greater and serum IL‐10, IL‐6, and CCL1 concentrations were significantly greater in patients with fatal disease than in patients with nonfatal disease; although the HLA-DR^+^CD86^+^ macrophage population was smaller in patients with fatal SFTS, and the population of CD163^+^CD206^+^ macrophages was lower in patients with fatal disease than in patients with nonfatal disease (**Figures 1a–c**).

**Figure 2:**
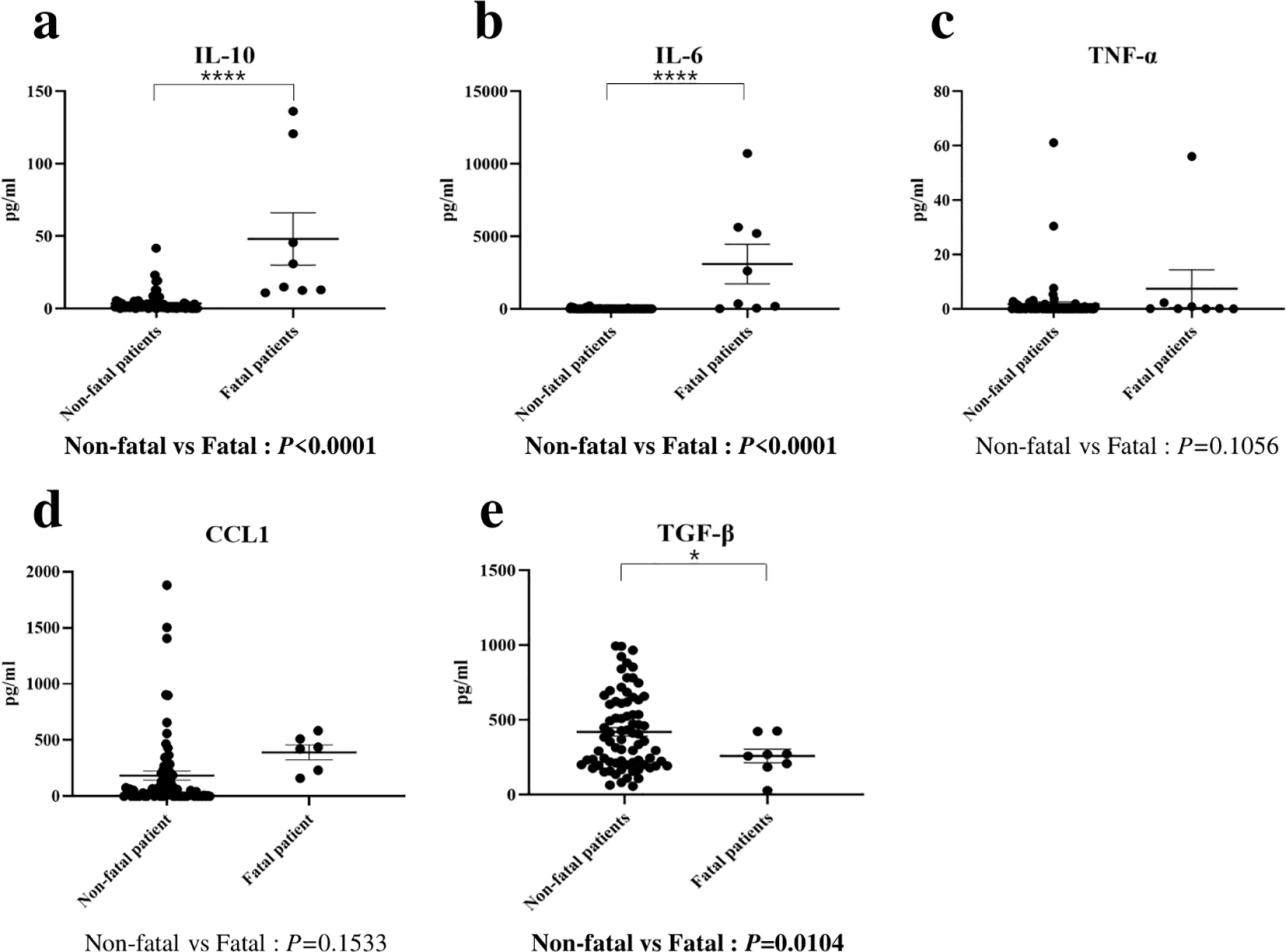
Serum levels of IL-10 **(a)**, IL-6 **(b)**, TNF-α **(c)**, CCL1 **(d)**, and TGF-β **(e)** in SFTS patients with nonfatal and fatal diseases from April 2013 to July 2024. The serum concentrations of IL-10, IL-6, TNF-α, CCL1, and TGF-β in SFTS patients after hospital admission were analyzed (**Supplementary Table 5**). Each dot shows the cytokine concentration in an individual, and the horizontal bars indicate the respective group median. SFTS, severe fever with thrombocytopenia syndrome. *: p < 0.05 / ****: p < 0.0001.

Among patients with nonfatal and fatal SFTS from May 2013 to July 2024, serum IL-10 (*p* < 0.0001), IL-6 (*p* < 0.0001), TNF-α (*p=*0.1056), and CCL1 (*p=*0.1533) concentrations were significantly higher in patients with fatal disease than in patients with nonfatal disease, and serum TGF-β (*p* = 0.0104) concentrations were significantly lower in the former group than in the latter group (**Figure 2**, **Supplementary Table 4**) (7, 8).

TGF-β1 signaling, which involves Smad3/P-Smad3 activation, leads to increased production of TGF-β1 through a positive feedback loop (15). Additionally, Smad3/P-Smad3 can stimulate the production of IL-10 (15).

Western blot analysis revealed that the expression of Smad3/P-Smad3 was markedly increased in patients with severe and fatal SFTS and that the expression of Smad3/P-Smad3 continued to increase in patients with fatal SFTS (**Figure 1c**).

JAK1/STAT3/Smad2 expression was not detected in patients with fatal disease, and there were no significant differences among patients with mild, moderate, and severe SFTS (**Figure 1c**).

These results suggest that Smad3/P-Smad3 is associated with high levels of IL-10, IL-6, and CCL1 in patients with fatal SFTS (**Figure 1c**). Moreover, Smad3^+^HLA-DR^hi^CD86^hi^ macrophages produce high amounts of IL-10, which can lead to increased IL-6 production and decreased TGF-β1 production, and these macrophages drive fatal cytokine storms in patients with fatal SFTS (8, 15).

We also suggest that the expansion of M2b-like macrophage populations could be induced in patients with fatal SFTS. M2b macrophages are characterized by high expression levels of HLA-DR and CD86 and a unique cytokine profile, which includes IL-6, IL-10, CCL1, and TNF-α (**Figures 1a, c**; **Supplementary Table 4**) (16, 17).

### SFTSV and SARS-CoV-2-infected and LPS-induced human macrophages: the role of IL-10 receptor blockade

We also studied the phenotypes of macrophages and the production of IL-10, IL-6, TNF-α, CCL1, and TGF-β during *in vitro* infection of human macrophages with SFTSV and SARS-CoV-2 and the induction of human macrophages with LPS and IL-10RA polyclonal antibodies.

The results revealed that the population of HLA-DR^+^CD86^+^ cells and the production of IL-6 and TNF-α decreased and that the population of CD163^+^CD206^+^ cells and the production of TGF-β increased.

**Figure 3:**
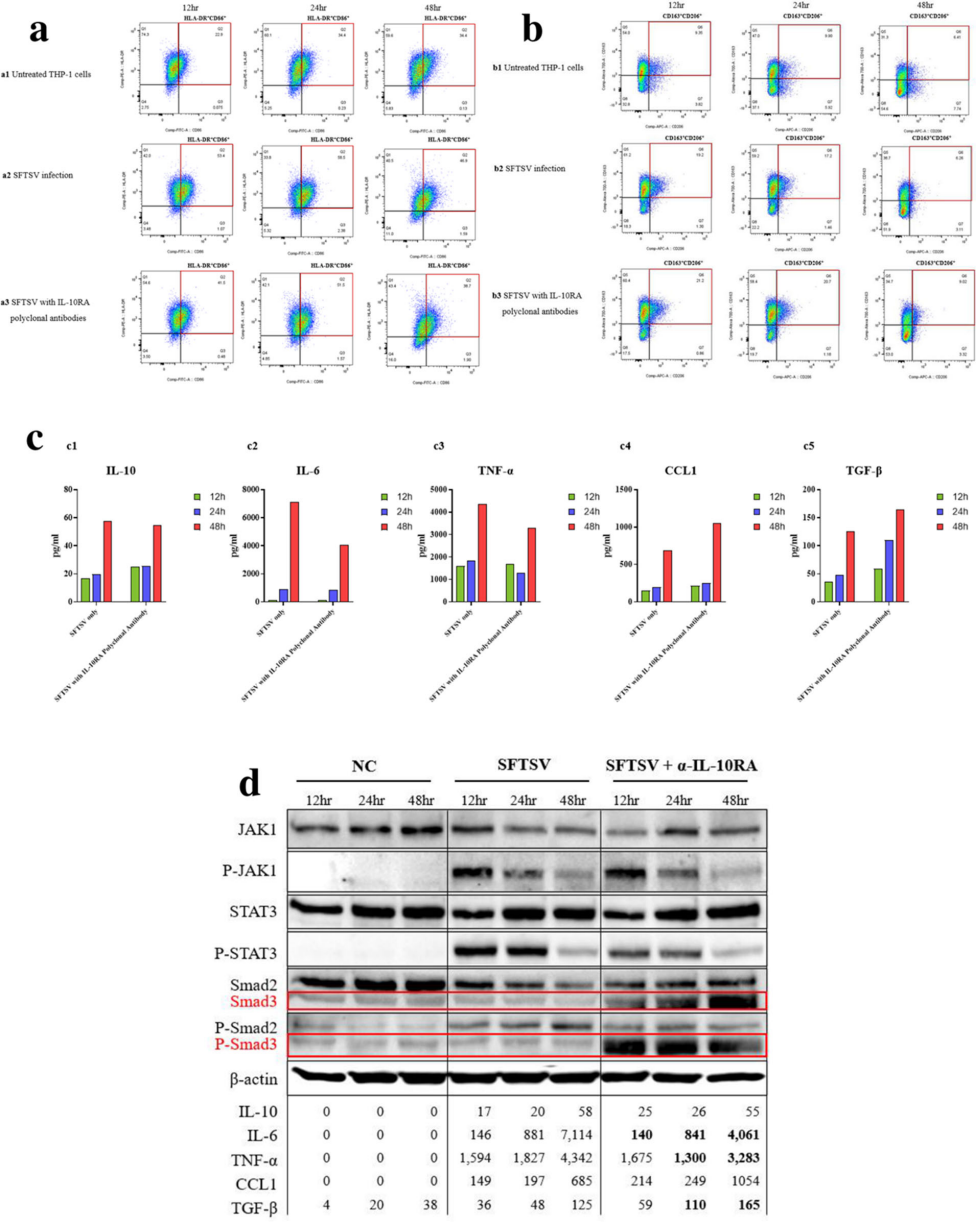
Multicolor flow cytometry analysis of HLA-DR and CD86 **(a1–a3)**, CD163 and CD206 **(b1–b3)**, the levels of IL‐10, IL‐6, TNF‐α, CCL-1, and TGF-β1 **(c1–c5)** and western blotting **(d1)** in SFTSV-infected THP-1 cells. Untreated THP‐1 cells **(a1, b1)**, THP-1 cells infected with SFTSV **(a2, b2)**, and SFTSV-infected THP-1 cells treated with anti-IL‐10RA polyclonal antibodies **(a3, b3)** are shown. The levels of IL-10 **(c1)**, IL-6 **(c2)**, TNF-α **(c3)**, CCL1 **(c4)**, and TGF-β **(c5)** were compared between THP-1 cells infected with SFTSV and SFTSV-infected THP-1 cells treated with IL‐10RA polyclonal antibodies. Western blotting **(d1)** was performed on untreated THP‐1 cells, THP-1 cells infected with SFTSV, and SFTSV-infected THP-1 cells treated with IL‐10RA polyclonal antibodies. The three groups were stimulated for 12 h (green), 24 h (blue), or 48 h (red). NC, negative control; SFTSV, severe fever with thrombocytopenia syndrome virus.

**Figure 4:**
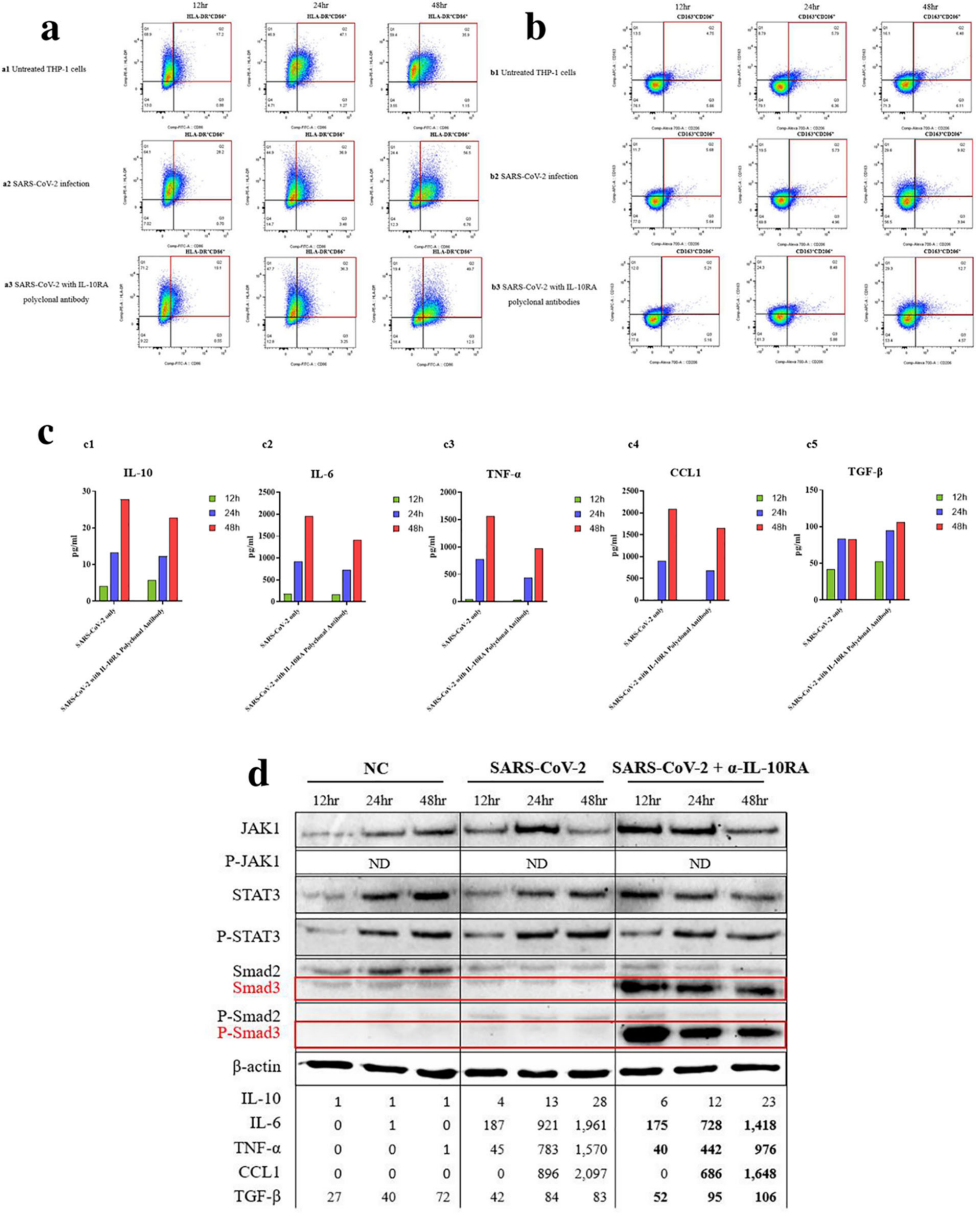
Multicolor flow cytometry analysis of HLA-DR and CD86 **(a1–a3)**, CD163 and CD206 **(b1–b3)**, the levels of IL‐10, IL‐6, TNF‐α, CCL-1, and TGF-β1 **(c1–c5)** and western blot **(d1)** of SARS-CoV-2-infected THP-1 cells. Untreated THP‐1 cells **(a1, b1)** and THP-1 cells were infected with SARS-CoV-2 **(a2, b2)**, and SARS-CoV-2-infected THP-1 cells were treated with the IL‐10RA polyclonal antibodies **(a3, b3)**. The levels of IL-10 **(c1)**, IL-6 **(c2)**, TNF-α **(c3)**, CCL1 **(c4)**, and TGF-β **(c5)** in THP-1 cells infected with SARS-CoV-2 and SARS-CoV-2-infected THP-1 cells treated with the IL‐10RA polyclonal antibodies were compared. Western blotting **(d1)** was performed on untreated THP‐1 cells, THP-1 cells infected with SARS-CoV-2, and SARS-CoV-2-infected THP-1 cells treated with anti-IL‐10RA polyclonal antibodies. The three groups were stimulated for 12 h (green), 24 h (blue), or 48 h (red). NC, negative control; SARS-CoV-2, severe acute respiratory syndrome coronavirus 2; α-IL-10RA, IL‐10RA polyclonal antibodies.

These findings suggested that treating macrophages with IL-10RA polyclonal antibodies could induce their transition to M2c macrophages, which are characterized by high expression of CD163 and CD206 and the production of IL-10 and TGF-β (**Figures 3a–c**; **4a–c**; **Supplementary Tables 5**–**7**) (15, 18).

**Figure 5:**
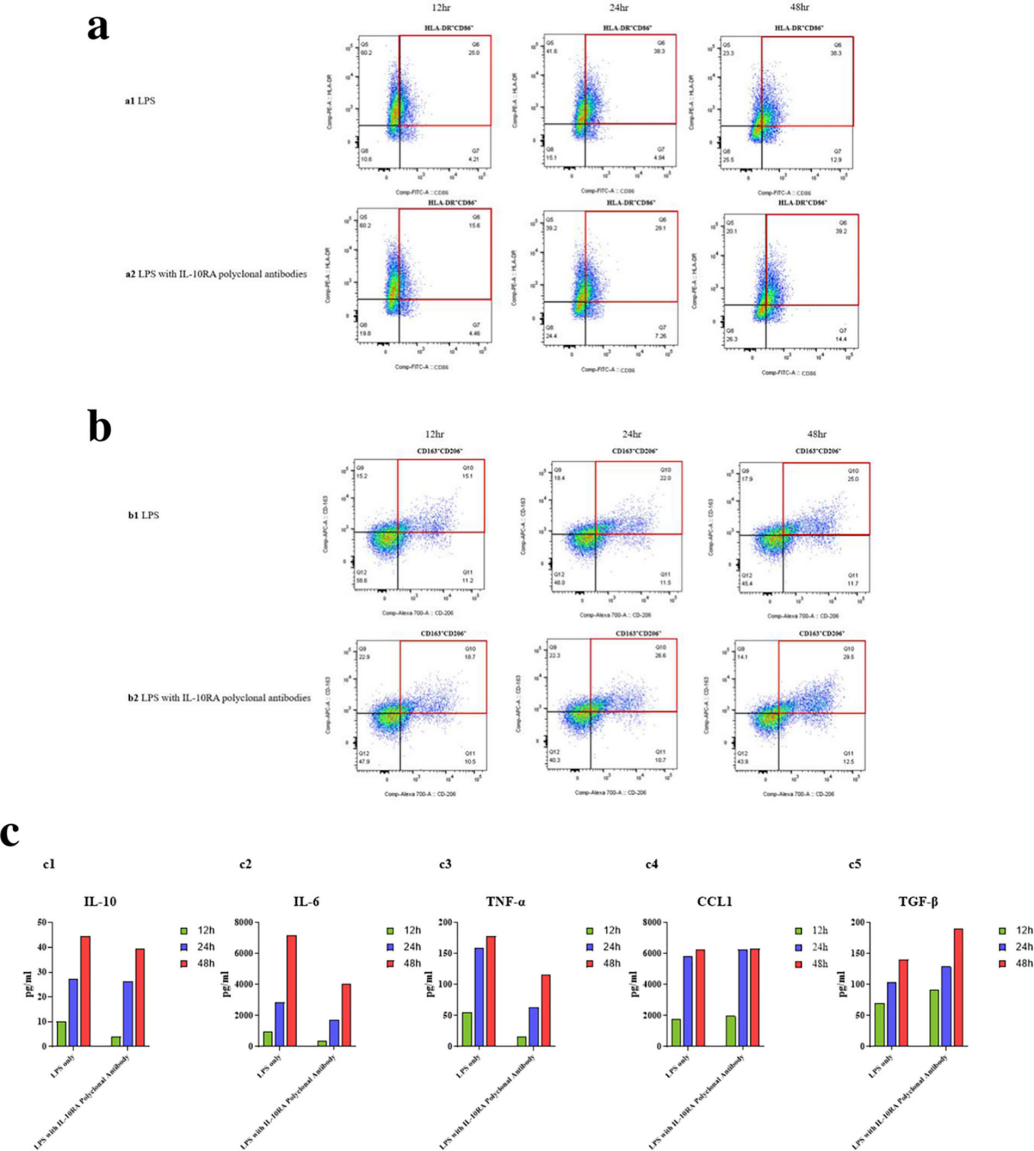
Multicolor flow cytometry analysis of HLA-DR and CD86 **(a1–a5)**, CD163 and CD206 **(b1–b5)**, and the levels of IL‐10, IL‐6, TNF‐α, CCL-1, and TGF-β1 **(c1–c5)** in LPS-induced THP-1-derived macrophages. THP-1 cells induced with LPS **(a1, b1)** and LPS-induced THP-1 cells treated with IL‐10RA polyclonal antibodies (A2 and B2) are shown. IL-10 **(c1)**, IL-6 **(c2)**, TNF-α **(c3)**, CCL1 **(c4)**, and TGF-β **(c5)** levels were compared between THP-1 cells induced with LPS and LPS-induced THP-1 cells treated with IL‐10RA polyclonal antibodies. The two groups were stimulated for 12 h (green), 24 h (blue), or 48 h (red). LPS, lipopolysaccharide.

Interestingly, Smad3/P-Smad3 expression was markedly upregulated in cells treated with the IL-10RA polyclonal antibody (**Figures 3d1**, **4d1**). The LPS-induced human macrophage results were similar to those for SFTSV- and SARS-CoV-2-infected cells treated with IL-10RA polyclonal antibodies: the population of HLA-DR^+^CD86^+^ macrophages and the levels of IL-6 and TNF-α decreased, and the population of CD163^+^ CD206^+^ macrophages and the levels of TGF-β increased (**Figures 5a–c**), while the level of Smad3/P-Smad3 was upregulated (**Supplementary Figure 1**).

However, there were no significant differences in CCL1 expression among SFTSV- and SARS-CoV-2-infected and LPS-induced human macrophages (**Figures 3c4**, **4c4**, **5c4**; **Supplementary Tables 5**–**7**).

### *In vitro* induction of human macrophages with serum from fatal SFTS patients: anti-IL-10RA monoclonal antibodies can reduce the production of IL10, IL-6, and the population of HLA-DR^+^CD86^+^ cells

**Figure 6:**
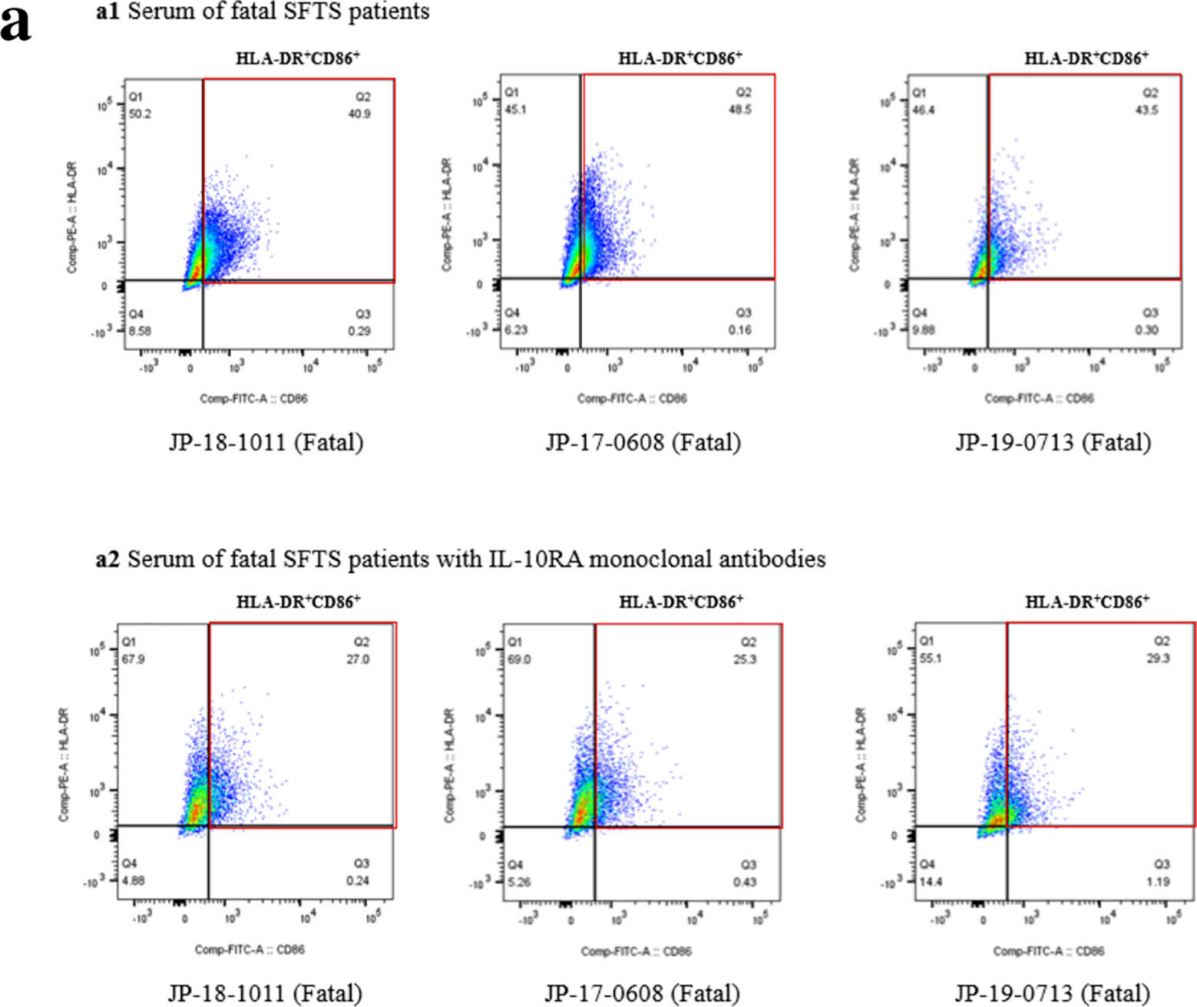
Multicolor flow cytometry analysis of HLA-DR and CD86 **(a1–a2)** in THP-1 cells treated with serum from patients with fatal SFTS. THP‐1 cells were incubated with serum from patients with fatal SFTS **(a1)** or serum plus IL‐10RA monoclonal antibodies **(a2)**. The two groups were stimulated for 24 h. Comparisons of the concentrations of IL-10, IL-6, TNF-α, CCL1, and TGF-β in patients with fatal SFTS are shown in **Supplementary Table 3**. SFTS, severe fever with thrombocytopenia syndrome; α-IL-10RA, IL‐10RA monoclonal antibodies.

When we treated human macrophages with serum from patients with fatal SFTS and IL-10RA monoclonal antibodies, the production of Il-10 and IL-6 and the population of HLA-DR^+^CD86^+^ cells decreased (**Table 2**, **Figure 6a**). These results showed that monoclonal antibodies against the IL-10 receptor are potential immune-based interventions against fatal SFTS (8, 11, 13).

## Discussion

Patients with fatal SFTS and severe/critical COVID-19 develop a pathological state termed CRS. In these patients, systemic inflammatory responses and hyperproduction of both IL-10 and IL-6 can generate a cytokine storm, suggesting that dramatic increases in IL-10 levels may play a pathological role in SFTS and severe/critical COVID-19 (7, 8, 11–13).

In this study, we revealed that a high population of HLA-DR^+^CD86^+^ macrophages can contribute to the hyperproduction of IL-10, IL-6, and TNF-α and the low production of TGF-β in SFTSV- and SARS‐CoV‐2‐infected and LPS‐induced human macrophages; moreover, a high CD163^+^CD206^+^ macrophage population contributes to low IL-10, IL-6, and TNF-α production and high TGF-β production when cells are treated with anti-IL-10RA polyclonal antibodies.

M2b macrophages express HLA-DR and CD86, which are also expressed on M1 macrophages; release high levels of IL-10; and produce proinflammatory cytokines such as IL-6 and TNF-α, along with the chemokine CCL1. These cells have multiple functions and are capable of both promoting and suppressing inflammation (16, 17).

M2c macrophages, a subtype of anti-inflammatory and alternatively activated macrophages, are characterized by high expression of the cell surface markers CD163 and CD206, as well as the secretion of IL-10 and TGF-β, and are critical for promoting anti-inflammatory responses and resolving inflammation and tissue damage within the body (18).

Therefore, we suggest that HLA-DR^+^CD86^+^ macrophages (M2b-like macrophages) can transition in response to blockade of IL-10 signaling. This shift from HLA-DR^+^CD86^+^ macrophages to CD163^+^CD206^+^ macrophages (M2c-like macrophages) is a biologically significant process, as M2c macrophages play a key role in immune regulation, including the anti-inflammatory response, through the secretion of TGF-β (18).

We also revealed that Smad3 contributes to pathological or protective effects during SFTSV and SARS-CoV-2 infection. Specifically, Smad3^+^ macrophages with high levels of HLA-DR and CD86 lead to pathological effects, whereas Smad3^+^ macrophages with high levels of CD163 and CD206 contribute to protective effector functions during SFTSV and SARS-CoV-2 infection.

A limitation of this study is that SFTS patients were admitted to a single tertiary hospital from May 2013 to July 2024, and THP-1 is a cell line isolated from the peripheral blood of an acute monocytic leukemia patient and may not perfectly replicate the behavior of primary human macrophages in complex disease states such as SFTS and COVID-19.

In conclusion, HLA-DR^hi^CD86^hi^ macrophages and IL-10 play important roles in the host immune response to fatal SFTSV and SARS-CoV-2 infection, and IL-10 could serve as a prognostic target. Antibodies targeting the IL-10 receptor are potential immune-based interventions against cytokine storms in patients with fatal SFTS and severe/critical COVID-19 (8, 11, 13). However, we also consider the potential risks of blocking IL-10 (an anti-inflammatory cytokine), such as exacerbating tissue damage or causing secondary infections.

## Data Availability

The original contributions presented in the study are included in the article/**Supplementary Material**. Further inquiries can be directed to the corresponding authors.

## Author disclaimer

Andrew G. Letizia (CAPT, MC, USN), Huy C. Nguyen (LCDR, MC, USN), and Hoai J. Hallam (LT, MSC, USN) are military Service members and employee of the U.S. Government. This work was prepared as part of his official duties at U.S. Naval Medical Research Unit INDO PACIFIC. Title 17 U.S.C. §105 provides that ‘Copyright protection under this title is not available for any work of the United States Government.’ Title 17 U.S.C. §101 defines a U.S. Government work as a work prepared by a military service member or employee of the U.S. Government as part of that person’s official duties.

## Supplementary material

Supplementary Figure 1 western blotting was performed on THP-1 cells induced with LPS and LPS-induced THP-1 cells treated with IL‐10RA polyclonal antibodies **(a, b)**. The two groups were stimulated for 12 h (green), 24 h (blue), or 48 h (red) **(a)**, and the two groups were stimulated for 24 h or 48 h **(b)**. LPS= lipopolysaccharide.
