# Supplementary material for: Antibodies against interleukin-10 receptor reduce IL-6 and TNF-α levels and increase TGF‐β levels in patients with severe fever with thrombocytopenia syndrome virus and SARS-CoV-2 infection
Source: Front Immunol. 2026 Jun 25;17:1828107. doi: 10.3389/fimmu.2026.1828107 (PMC13347219; doi:10.3389/fimmu.2026.1828107)
Supplement: Supplementary Figure 1 — western blotting was performed on THP-1 cells induced with LPS and LPS-induced THP-1 cells treated with IL‐10RA polyclonal antibodies (a, b). The two groups were stimulated for 12 h (green), 24 h (blue), or 48 h (red) (a), and the two groups were stimulated for 24 h or 48 h (b). LPS= lipopolysaccharide. [file Table1.docx]

**Supplemental Table 1** Baseline demographic and clinical characteristics of patients with laboratory-confirmed SFTS from May 2013 to July 2024.

| Variables | All patients (*n*=86) | Severity | | | |
| --- | --- | --- | --- | --- | --- |
|  |  | mild (*n*=53) | moderate (*n*=20) | severe (*n*=13) | *p* value |
| Age, years | 63.3 ± 13.6 | 59.7 ± 14.3 | 73.0 (63.3–78.8) | 66.0 (61.5–72.0) | 0.028 |
| Male gender, n (%) | 49 (57.0) | 30 (56.6) | 11 (55.0) | 8 (61.5) | 0.930 |
| CCI (score) | 0.5 ± 0.7 | 0 (0–1.0) | 0.5 (0.0–1.0) | 0 (0–2.0) | 0.110 |
| Tick bite lesion, n (%) | 32 (37.2) | 23 (43.4) | 5 (25.0) | 4 (30.8) | 0.305 |
| From onset of disease to admission (d) | 4.4 ± 2.3 | 4.0 (3.0–5.0) | 4.5 (2.3–7.0) | 4.0 (3.0–4.5) | 0.259 |
| From diagnosis to admission (d) | 1.2 ± 1.2 | 1.0 (0–2.0) | 1.0 (0–3.0) | 1.0 (0.3–1.8) | 0.511 |
| Fever, peak (within 24 hours) | 38.5 ± 0.8 | 38.2 (38.0–39.0) | 38.6 (38.0–39.0) | 38.5 (38.0–39.0) | 0.953 |
| Duration of fever (d) | 4.2 ± 3.0 | 2.0 (1.0–3.0) | 3.0 (2.0–5.0) | 5.0 (2.3–8.8) | 0.247 |
| Laboratory findings |  |  |  |  |  |
| WBC (cells/μL) | 2342 ± 1683 | 1800 (1200–2500) | 1800 (1600–3475) | 2000 (1630–4150) | 0.634 |
| ANC (cells/μL) | 1354 ± 1258 | 918 (540–1592) | 1100 (635–2581) | 1230 (638–1610) | 0.506 |
| Lymphocyte fraction (%) | 36.2 ± 17.0 | 36.0 (23.0–49.7) | 30.5 (16.5–51.0) | 38.0 (24.5–44.5) | 0.368 |
| Platelet (×10^3^cells/μL) | 75.9 ± 38.0 | 85.0 (55.5–114.5) | 47.0 (35.8–77.3) | 55.0 (38.5–81.5) | 0.012 |
| CRP (mg/dL) | 1.1 ± 2.1 | 0.2 (0.0–1.0) | 0.3 (0–1.0) | 0.8 (0.2–5.0) | 0.098 |
| aPTT (sec) | 41.7 ± 11.4 | 38.0 (35.0–43.0) | 42.0 (33.0–51.0) | 49.0 (36.5–64.5) | 0.141 |
| Total bilirubin (mg/dL) | 0.2 ± 0.4 | 0.0 (0.0–0.3) | 0.0 (0.0–0.4) | 0.2 (0.0–1.0) | 0.639 |
| AST (IU/L) | 218 ± 307 | 80.0 (53.0–148.5) | 148.0 (71.5–351.8) | 219 (71.5–667.5) | 0.119 |
| ALT (IU/L) | 93 ± 100 | 57.0 (29.5–97.5) | 61.0 (40.5–171.5) | 84.0 (28.0–168.0) | 0.650 |
| Cr (mg/dL) | 1.4 ± 0.5 | 1.0 (1.0–1.0) | 1.0 (1.0–1.3) | 1.0 (1.0–2.0) | 0.041 |
| LDH (IU/L) | 976 ± 1104 | 582 (412–775) | 874 (543–1754) | 1276 (633–2059) | 0.006 |
| CPK (IU/L) | 1166 ± 2518 | 254 (123–883) | 293 (198–1748) | 588 (345–4377) | 0.010 |
| Ferritin (ng/ml) | 1093 ± 755 | 878 (342–2000) | 643 (438–1853) | 2000 (464–2000) | 0.814 |
| Initial SFTS M-segment Ct value | 28.6 ± 3.2 | 29.4 (27.7–30.9) | 30.0 (28.3–30.2) | 26.9 (24.9–28.9) | 0.060 |
| Initial SFTS S-segment Ct value | 27.9 ± 3.3 | 28.7 (26.5–30.3) | 29.3 (28.5–30.2) | 26.4 (24.1–27.8) | 0.011 |
| Initial SFTS viral load, copies RNA/ml | 65778256 ± 573720466 | 58579 (4736–306788) | 206499 (3474–2517082) | 143948 (8735–8015756) | 0.260 |
| Interleukin-6, pg/ml (initial) | 281.0 ± 1709.0 | 10.0 (6.0–18.5) | 13.5 (8.0–41.0) | 61.0 (25.0–1377) | 0.007 |
| Type of treatment, n (%) |  |  |  |  |  |
| Conservative care | 48 (55.8) | 37 (69.8) | 9 (45.0) | 2 (15.4) | 0.004 |
| TPE | 29 (33.7) | 11 (20.8) | 9 (45.0) | 9 (69.2) | 0.002 |
| Tocilizumab | 8 (9.3) | 5 (9.4) | 2 (10.0) | 1 (7.7) | 0.941 |
| IVIG | 1 (1.2) | 0 | 0 | 1 (7.7) | 0.122 |
| From admission to treatment (d) | 1.9 ± 1.4 | 2.0 (1.0–3.0) | 1.5 (1.0–3.3) | 1.0 (0.5–3.0) | 0.007 |
| Inotropic drug, n (%) | 13 (15.1) | 0 | 2 (10.0) | 11 (84.6) | <0.001 |
| Mechanical ventilation, n (%) | 9 (10.5) | 0 | 0 | 9 (69.2) | <0.001 |
| Renal replacement treatments, n (%) | 7 (8.1) | 0 | 0 | 7 (53.8) | <0.001 |
| Length of hospitalization (d) | 8.8 ± 5.6 | 7.0 (5.0–8.0) | 10.5 (7.0–12.8) | 9.0 (4.0–22.0) | 0.004 |
| Fatality, n (%) | 12 (14.0) | 0 | 0 | 12 (92.3) | <0.001 |
| 14-day mortality, n (%)  28-day mortality, n (%) | 6 (7.0)  8 (9.3) | 0  0 | 0  0 | 6 (46.2)  8 (61.5) | <0.001  <0.001 |

Values are presented as mean ± standard deviation, median (interquartile range), or n (%). *P* values are derived from Kruskal-Wallis test for continuous data and chi-square test for categorical data between each group. n, number CCI, comorbidity index score (calculated by Charlson comorbidity index); d, days; WBC, white blood cell; ANC, absolute neutrophil count; CRP, C-reactive protein; aPTT, activated partial thromboplastin time; AST, aspartate aminotransferase; ALT, alanine aminotransferase; Cr, creatinine; LDH, lactate dehydrogenase; CPK, creatinine phosphokinase; TPE, therapeutic plasma exchange; TCZ, tocilizumab; IVIG, immunoglobulin G.

**Supplemental Table 2** Comparison of IL-10, IL-6, TNF-α, CCL1, and TGF-β concentrations of fatal SFTS patients.

| SFTS patients | Age (years)/Sex | Date | Outcome | IL-10 | IL-6 | TNF-α | CCL1 | TGF-β |
| --- | --- | --- | --- | --- | --- | --- | --- | --- |
| JP-18-1011 | 68/M | 2018-10-11 | Fatal | 120.73 | 5203.18 | 56.00 | 233.25 | 422.83 |
| JP-17-0608 | 64/M | 2017-06-08 | Fatal | 30.90 | 10717.59 | 0.00 | 160.43 | 269.83 |
| JP-19-0713 | 92/F | 2019-07-13 | Fatal | 14.98 | 354.36 | 0.15 | 584.27 | 258.55 |

Unit: pg/mL.

**Supplemental Table 3** Clinical characteristics of SFTS patients.

| No. | Age  (year/sex) | Diagnosis  (YY/MM/DD) | Severity | Symptom onset - diagnosis (day) | Exposure type | CCI score | ANC  (cells/μL) | Platelet (cells/μL) | AST/ALT (IU/L) | Cr (mg/dL) | CPK (IU/L) | LDH (IU/L) | aPTT (sec) | MODS  (initial) | MODS (peak) | SFTSV viral load copies RNA/mL | Type of treatment | Length of hospital stay (day) |
| --- | --- | --- | --- | --- | --- | --- | --- | --- | --- | --- | --- | --- | --- | --- | --- | --- | --- | --- |
| JP-23-0707 | 49/F | 2023/07/06 | Severe (F) | 4 | Wild cat | 0 | 390 | 7000 | 41/25 | 0.7 | 72 | 199 | 35 | 4 | 14 | 4,735,471 | TPE, TCZ | 6 |
| JP-24-0708-2 | 66/M | 2024/07/08 | Severe (R) | 4 | Tick bite | 2 | 1009 | 25000 | 622/236 | 2.74 | 19292 | 1529 | 49 | 5 | 9 | 2109644 | TPE | 22 |
| JP-24-0708-1 | 64/M | 2024/07/07 | Mild (R) | 9 | Tick bite | 2 | 379 | 40000 | 191/124 | 1.12 | 1159 | 777 | 43 | 3 | 3 | 2394 | Cons | 7 |
| JP-24-0715-1 | 78/M | 2024/07/15 | Mild (R) | 5 | Tick bite | 3 | 1710 | 10100 | 450/133 | 1.91 | 4111 | 1438 | 45 | 3 | 4 | 594 | Cons | 5 |
| JP-24-0715-2 | 49/M | 2024/07/15 | Mild (R) | 6 | Tick bite | 0 | 690 | 45000 | 149/74 | 0.72 | 367 | 581 | 45 | 3 | 3 | 7468 | Cons | 5 |

No. number of patients; M, male; F, female; YY/MM/DD, years/months/days; D, death; R, recovery; CCI, comorbidity index score (calculated by Charlson comorbidity index); ANC, absolute neutrophil count; AST, aspartate aminotransferase; ALT, alanine aminotransferase; Cr, creatinine; LDH, lactate dehydrogenase; CPK, creatinine phosphokinase; aPTT, activated partial thromboplastin time; MODS, multiple organ dysfunction score; SFTSV, severe fever with thrombocytopenia syndrome virus, TPE, therapeutic plasma exchange; TCZ, tocilizumab; Cons, conservative care; N/A, not applicable; F, fatal; R, recovery.

**Supplemental Table 4** Comparison of IL-10, IL-6, TNF-α, CCL1, and TGF-β concentrations between patients with nonfatal and fatal disease from May 2013 to July 2024.

| Characteristic | Patients with nonfatal disease  (*n*=77) | Patients with fatal patients  (*n*=8) | *p*-value |
| --- | --- | --- | --- |
| **IL-10** | 3.56 (0.00 - 41.68) | **48.10 (10.90 - 136.16)** | **<0.0001** |
| **IL-6** | 13.96 (0.00 - 214.93) | **3098.17 (14.23 - 10717.59)** | **<0.0001** |
| TNF-α | 1.74 (0.0 - 61.07) | 7.43 (0.0 - 56.00) | 0.1056 |
| CCL1 | 183.71 (0.0 - 1880.71) | 391.48 (160.43 - 584.27) | 0.1533 |
| **TGF-β** | 419.35 (56.90 - 993.99) | **258.78 (28.72 - 425.99)** | **0.0104** |

Unit: pg/mL.

**Supplemental Table 5** Comparison of IL-10, IL-6, TNF-α, CCL1, and TGF- β between SFTSV only and SFTSV with IL-10RA polyclonal antibodies.

| Sample | Time (Hours) | IL-10 | **IL-6** | **TNF-α** | CCL1 | **TGF- β** |
| --- | --- | --- | --- | --- | --- | --- |
| SFTSV only | 12 | 16.72 | 145.70 | 1594.16 | 149.17 | 35.67 |
| SFTSV only | 24 | 19.75 | 881.26 | 1826.57 | 196.99 | 47.78 |
| SFTSV only | 48 | 57.71 | 7113.96 | 4342.28 | 684.87 | 125.10 |
| SFTSV with IL-10RA polyclonal antibodies | 12 | 24.95 | **139.94** | 1675.11 | 213.90 | 58.99 |
| SFTSV with IL-10RA polyclonal antibodies | 24 | 25.53 | **840.54** | **1300.42** | 248.85 | **110.05** |
| SFTSV with IL-10RA polyclonal antibodies | 48 | 54.51 | **4061.02** | **3282.70** | 1053.85 | **164.83** |

Unit: pg/mL.

**Supplemental Table 6** Comparison of IL-10, IL-6, TNF-α, CCL1, and TGF- β between SARS-CoV-2 only and SARS-CoV-2 with IL-10RA polyclonal antibodies.

| Sample | Time (Hours) | IL-10 | **IL-6** | **TNF-α** | **CCL1** | **TGF-β** |
| --- | --- | --- | --- | --- | --- | --- |
| SARS-CoV-2 only | 12 | 4.19 | 187.28 | 44.90 | 0 | 41.95 |
| SARS-CoV-2 only | 24 | 13.31 | 921.45 | 783.32 | 896.26 | 83.75 |
| SARS-CoV-2 only | 48 | 27.79 | 1960.92 | 1570.06 | 2097.12 | 82.55 |
| SARS-CoV-2 only with IL-10RA polyclonal antibodies | 12 | 5.77 | **174.51** | **40.49** | 0 | **52.47** |
| SARS-CoV-2 only with IL-10RA polyclonal antibodies | 24 | 12.32 | **728.30** | **441.95** | **685.98** | **94.63** |
| SARS-CoV-2 only with IL-10RA polyclonal antibodies | 48 | 22.74 | **1418.48** | **975.59** | **1648.13** | **106.14** |

Unit: pg/mL.

**Supplemental Table 7** Comparison of IL-10, IL-6, TNF-α, CCL1, and TGF- β concentrations between LPS only and LPS with IL-10RA polyclonal antibodies.

| Sample | Time (Hours) | IL-10 | **IL-6** | **TNF-α** | CCL1 | **TGF- β** |
| --- | --- | --- | --- | --- | --- | --- |
| LPS only | 12 | 10.03 | 953.67 | 54.90 | 1748.71 | 69.68 |
| LPS only | 24 | 27.22 | 2855.68 | 159.46 | 5840.23 | 104.32 |
| LPS only | 48 | 44.47 | 7165.68 | 178.23 | 6237.47 | 140.45 |
| LPS with IL-10RA polyclonal antibodies | 12 | 4.04 | **390.97** | **15.91** | 1966.90 | **92.00** |
| LPS with IL-10RA polyclonal antibodies | 24 | 26.16 | **1708.43** | **63.61** | 6252.29 | **128.79** |
| LPS with IL-10RA polyclonal antibodies | 48 | 39.40 | **4034.26** | **115.19** | 6312.12 | **189.82** |

Unit: pg/mL.
