# Supplementary figures and images for: Antibodies against interleukin-10 receptor reduce IL-6 and TNF-α levels and increase TGF‐β levels in patients with severe fever with thrombocytopenia syndrome virus and SARS-CoV-2 infection
Source: Front Immunol. 2026 Jun 25;17:1828107. doi: 10.3389/fimmu.2026.1828107 (PMC13347219; doi:10.3389/fimmu.2026.1828107)

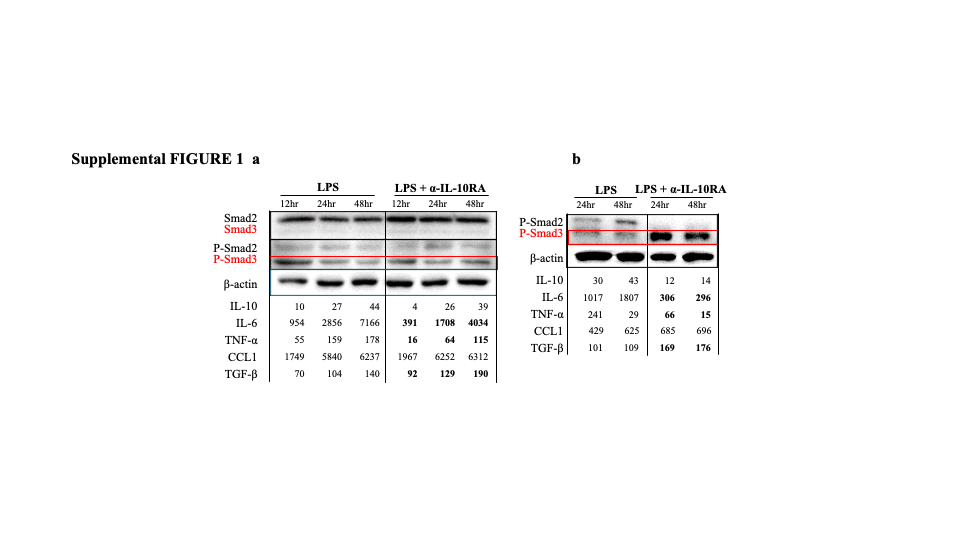

Supplement: Supplementary file 2 [file Image1.tiff]
